# Deep learning and ensemble stacking technique for differentiating polypoidal choroidal vasculopathy from neovascular age-related macular degeneration

**DOI:** 10.1038/s41598-021-86526-2

**Published:** 2021-03-30

**Authors:** Yu-Bai Chou, Chung-Hsuan Hsu, Wei-Shiang Chen, Shih-Jen Chen, De-Kuang Hwang, Yi-Ming Huang, An-Fei Li, Henry Horng-Shing Lu

**Affiliations:** 1 Department of Ophthalmology, Taipei Veterans General Hospital, Taipei, Taiwan; 2 School of Medicine, National Yang Ming Chiao Tung University, Taipei, Taiwan; 3 Institute of Statistics, National Yang Ming Chiao Tung University, Hsin Chu, Taiwan; 4 Department of Ophthalmology, Cheng Hsin General Hospital, Taipei, Taiwan

**Keywords:** Medical research, Mathematics and computing

## Abstract

Polypoidal choroidal vasculopathy (PCV) and neovascular age-related macular degeneration (nAMD) share some similarity in clinical imaging manifestations. However, their disease entity and treatment strategy as well as visual outcomes are very different. To distinguish these two vision-threatening diseases is somewhat challenging but necessary. In this study, we propose a new artificial intelligence model using an ensemble stacking technique, which combines a color fundus photograph-based deep learning (DL) model and optical coherence tomography-based biomarkers, for differentiation of PCV from nAMD. Furthermore, we introduced multiple correspondence analysis, a method of transforming categorical data into principal components, to handle the dichotomous data for combining with another image DL system. This model achieved a robust performance with an accuracy, sensitivity, specificity, and area under the receiver operating characteristic curve of 83.67%, 80.76%, 84.72%, and 88.57%, respectively, by training nearly 700 active cases with suitable imaging quality and transfer learning architecture. This work could offer an alternative method of developing a multimodal DL model, improve its efficiency for distinguishing different diseases, and facilitate the broad application of medical engineering in a DL model design.

## Introduction

Polypoidal choroidal vasculopathy (PCV) is currently considered a subtype of pachychoroid spectrum disease with clinical imaging features similar to those of typical neovascular age-related macular degeneration (nAMD)^1,2^. Although nAMD and PCV have some similarity in clinical imaging manifestations, their disease entities, treatment strategies as well as outcomes are different. Eyes with PCV are more prone to manifesting massive subretinal hemorrhage, recurrent hemorrhagic/serous pigment epithelial detachment (PED), or breakthrough vitreous hemorrhage compared to nAMD^3,4^. Therefore, to distinguish these two vision-threatening diseases is somewhat challenging but necessary. The gold standard examination to differentiate between nAMD and PCV is indocyanine green angiography (ICGA)^5^. According to the EVEREST criteria^6^ and Japanese Study Group Guidelines^7^, both color fundus photographs (CFPs) and ICGA are essential for diagnosing PCV. However, ICGA is a time-consuming, invasive image examination, and unavailable in some hospitals.

It is still a controversy whether PCV is a different entity from nAMD or is a subtype of nAMD with polypoidal(aneurysmal) changes. Several studies have focused on non-ICGA imaging analyses to differentiate PCV from nAMD, including CFPs, spectral-domain optical coherence tomography (OCT), enface OCT, OCT angiography, autofluorescence, fluorescence angiography (FA), or even multimodal and multicolor imaging analysis^1,8–12^. However, in real-world clinical practice, the effective and convenient tool for differentiating PCV from nAMD is still lacking.

Artificial intelligence (AI) has been applied widely in several medical imaging fields, including ophthalmology^13,14^. After advancements in computer calculating power and graphic processing units, deep learning (DL) techniques were developed to exploit complex imaging information for improved learning performance. DL can use multiple processing layers to learn representative imaging data and further to obtain the diagnostic output^15^. Even though single-modal imaging analysis using ICGA to differentiate PCV from AMD have reported well results^16^, it is still limited by the exam process and the accessibility to ICGA. Other studies that attempted to distinguish PCV using multi-modal image have yielded good results^9,17^. However, multi-modal imaging analysis remains challenging, it still worth the effort to develop a novel DL model for clinical practice.

In this study, we combine CFPs and OCT biomarkers by applying Google’s EfficientNet^18^ and demonstrate the feasibility and efficacy of this novel DL model by stacking technique to distinguish between PCV and AMD.

## Results

**Figure 1:**
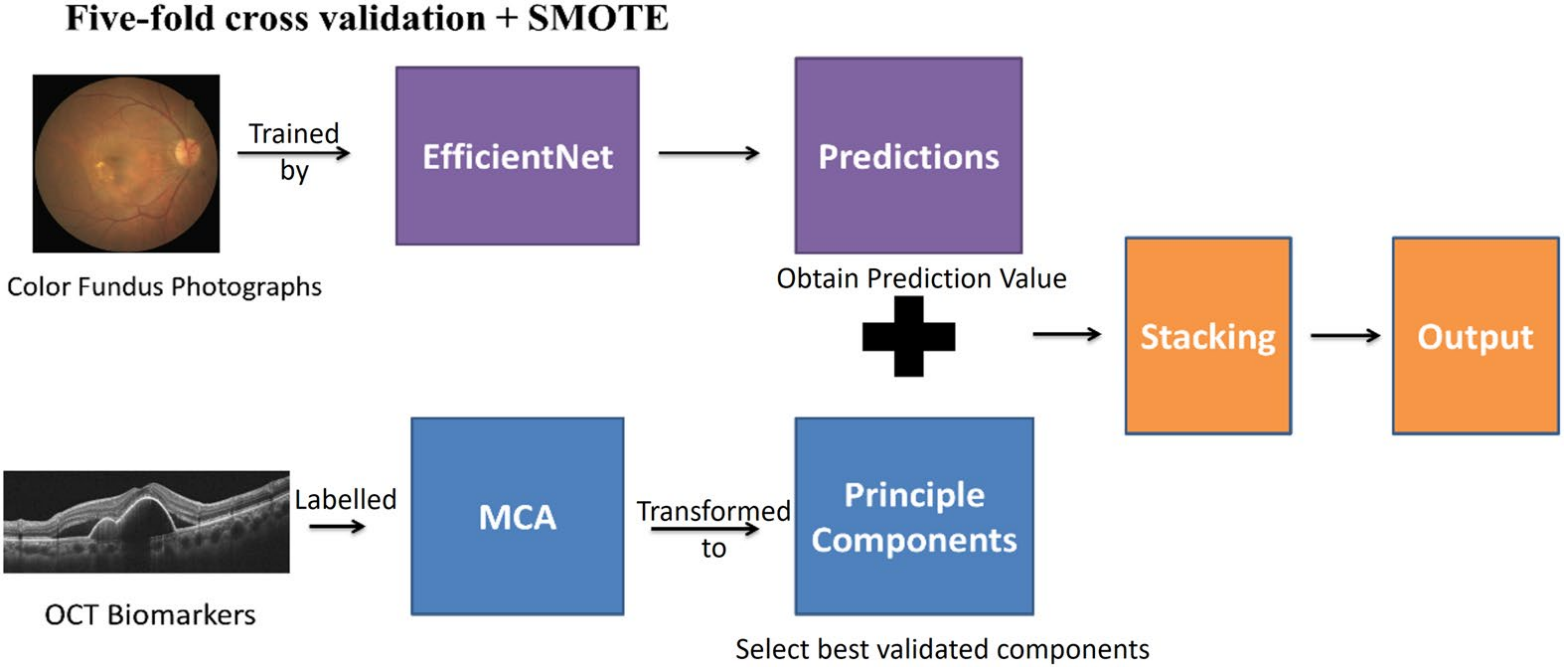
Framework of model training. The flowchart revealed the framework of model training by combining CFPs and OCT biomarkers. First, the CFPs were trained by EfficientNet model to obtain prediction value. Second, the OCT biomarkers were labelled by retinal specialist and then were dimension-reduced and transformed to principal components by multiple correspondence analysis (MCA). Third, merging the two steps to achieve the final results by stacking technique. Abbreviation: *MCA* multiple correspondence analysis.

In total, 491 nAMD cases and 208 PCV cases were confirmed and enrolled for model training. We combined CFPs and OCT biomarkers and exploited a new prediction model using a three-step method (Fig. 1) to differentiate PCV from nAMD.

### **Step 1**

CFP-based model.

**Figure 2:**
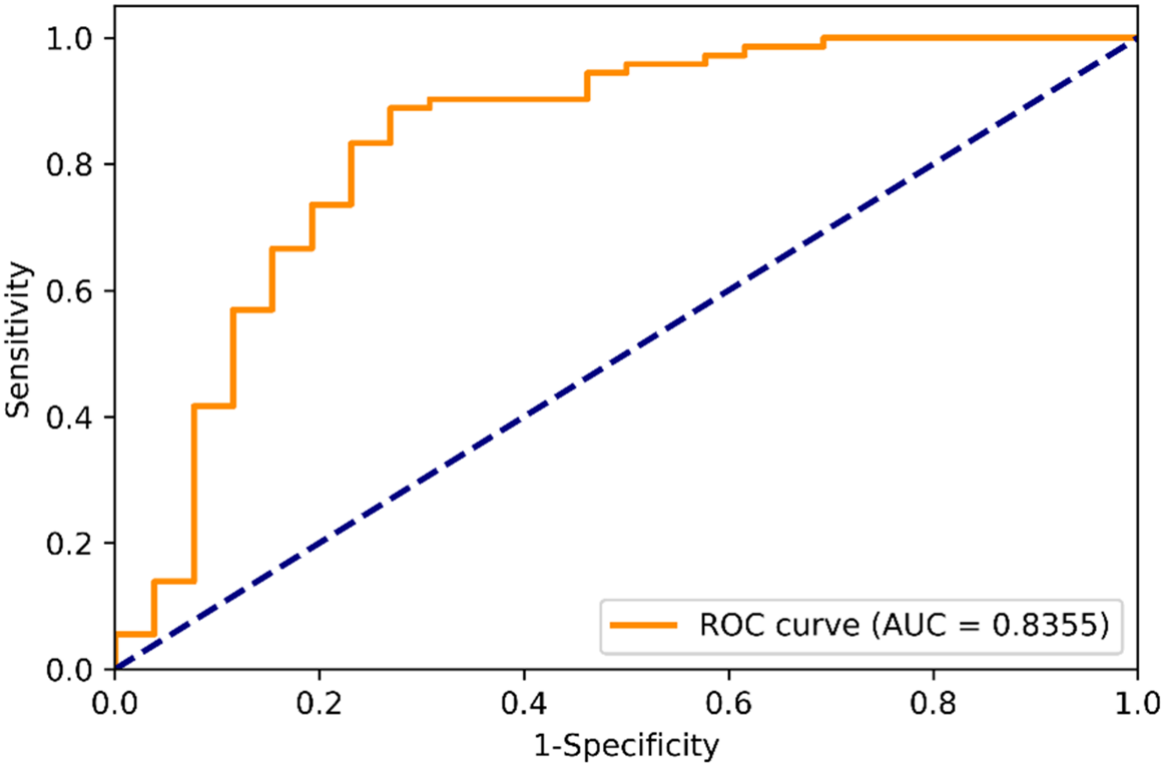
ROC curve in CFPs-based model. The area under curve (AUC) in this EfficientNet model by CFPs was 83.55%.

In the first step, we trained the model using only CFPs in EfficientNet-B3^18^ and validated the model. The validation performance is shown in Table 1. The standard deviation for accuracy is 0.0076, demonstrating that the model prediction is stable for each validation set. The prediction performance of the testing dataset is shown in Table 2 and Fig. 5. The accuracy, sensitivity, specificity are 77.55%, 76.92%, and 77.77%, respectively. The area under the receiver operating characteristic (ROC) curve (AUC) is 83.55% (Fig. 2).

**Table 1:** Validation performance of CFPs-based model.

Average accuracy	Standard deviation of accuracy	Average AUC
0.8070	0.0076	0.8355

In the first step of this study, we trained and validated the model only with color fundus photographs (CFPs).

**Table 2:** Prediction performance of testing dataset in CFPs-based model.

Accuracy	Sensitivity	Specificity
0.7755	0.7692	0.7777

The performance in testing was around 77% in each evaluation metrics.

### **Step 2**

OCT biomarkers conversion.

**Figure 3:**
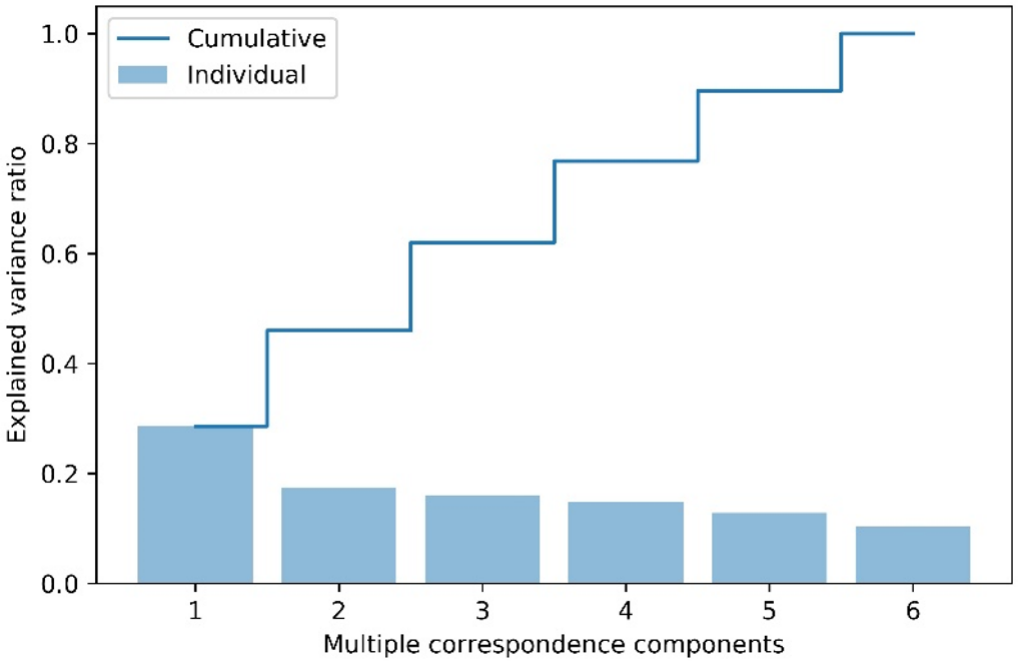
Conversion of categorical OCT biomarkers into different numbers of MCA components. The y axis showed the explained variance ratio, whereas the x axis showed the number of multiple correspondence components. It revealed that the first four components possessed nearly 80% explained variation.

In the second step, we converted the six specific OCT biomarkers, , including the double-layer sign, triple-layer sign, thumb-like PED, notch PED, M-shape PED, and ring/bubble sign (including strings-of-pearl sign)^1^*,* into continuous principal components through multiple correspondence analysis (MCA). The characteristic distribution of OCT biomarkers in PCV and nAMD are listed in Table 3. After transformation through MCA, the principal components were presented in a matrix format and considered extracted features for further model training. The Fig. 3 demonstrates the converted OCT biomarkers as different numbers of MCA components and the corresponding explainable variance. The selection of MCA and the effect of variable number of MCA components are listed in Supplementary Table S2.

**Table 3:** Characteristic distribution of OCT biomarkers in PCV and nAMD subjects.

Group	Biomarkers					
	Double layer sign (%)	Triple layer sign (%)	Notch-PED (%)	M-shape PED (%)	Thumb sign (%)	Bubble/ring sign (including strings-of-pearl sign) (%)
PCV (208 eyes)	78.5	59.2	25	15.4	7.3	7.3
nAMD (491 eyes)	45.2	35.6	13.2	2.8	10	1

This table revealed the characteristic distribution of OCT biomarkers in nAMD and PCV group.

### **Step 3**

Stacking technique for combining into new features.

**Figure 4:**
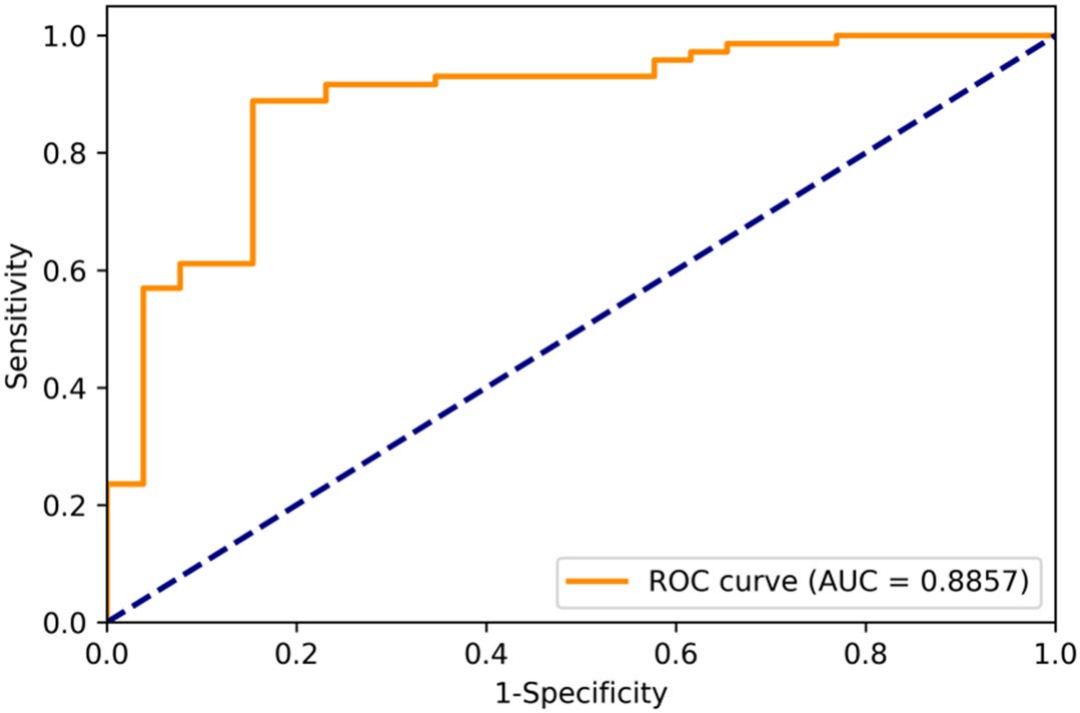
ROC curve of final prediction model in combination of CFPs and OCT biomarkers. The area under curve (AUC) showed 88.57%.

In the final step, we combined the converted OCT biomarkers (as MCA components) and CFPs (as prediction value) into new features for further model training. The first four MCA components were selected because of the best validating performance and well-explained variance. Table 3 demonstrates the result of the stacking technique by combining CFPs with selected MCA components from OCT biomarkers. After combining these two features, the performance can approach up to 83.67% in accuracy, 80.76% in sensitivity, and 84.72% in specificity, with an AUC of 88.57% (Table 4 and Fig. 4).

**Table 4:** Comparison of prediction performance in testing dataset between CFP-based model and the model combining CFPs and OCT biomarkers with stacking technique.

Model	Accuracy	Sensitivity	Specificity
CFP-based	0.7755	0.7692	0.7777
CFP + OCT biomarkers	0.8367	0.8076	0.8472

After combining OCT biomarkers with stacking technique, the testing performance can improved to 83.67% in accuracy, 80.76% in sensitivity, and 84.72% in specificity.

## Discussion

There is no doubt that the multi-modal DL model has draw more attention in the development of medical AI. For example, Xu et al. proposed a bi-modal DL model directly combining entire CFPs and OCT images with robust results to differenciate PCV from AMD^17^. The present study introduced a novel bi-modal DL model for clinical ophthalmologists, which combined the DL algorithm (to obtain prediction values of CFPs) and MCA transformation data (from categorical OCT biomarkers) using an ensemble stacking technique to classify PCV and nAMD. We confirmed the feasibility and efficacy of this training model, with the accuracy rate: 83.67%, sensitivity 80.76%, specificity 84.72%, AUC 88.57%. To our knowledge, this article, for the first time, presents a DL training model with an ensemble stacking technique, combining CFPs and clinical features of OCT biomarkers, to distinguish between PCV and nAMD.

**Figure 5:**
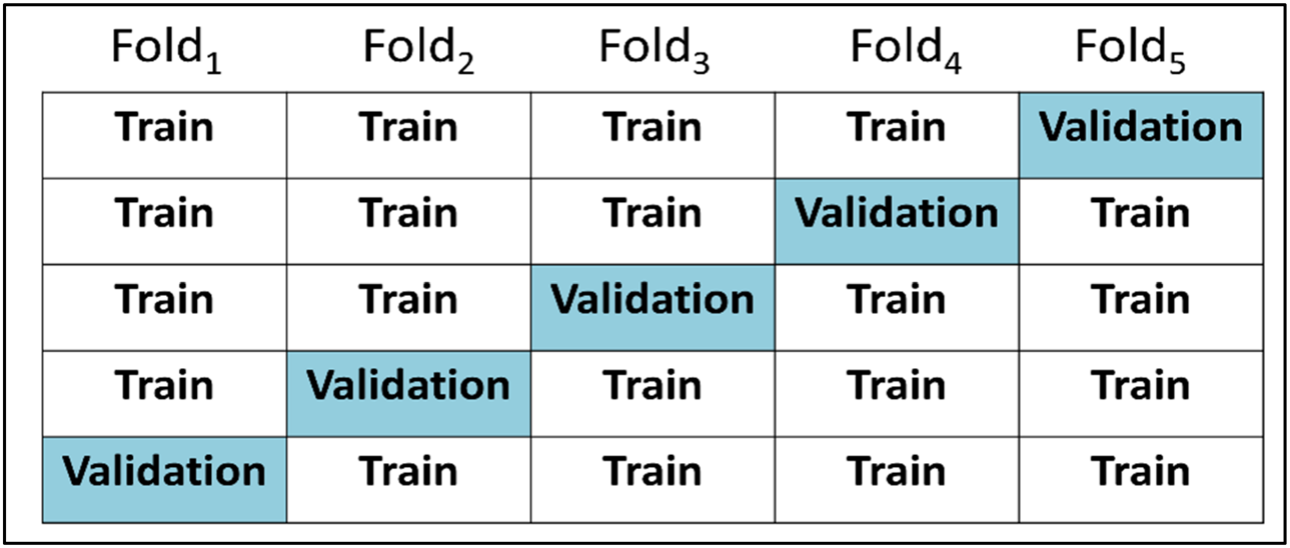
Five-fold cross-validation. This figure showed the way of five-fold cross-validation and how the dataset was split into five subsets.

In the present study, the CFP-based model alone had already reached an accuracy of 77.6% and AUC of 83.5% using a deep learning model (Table 2 and Fig. 5). Gemmy Cheung et al. recently summarized the consensus of diagnostic criteria from the Asia Pacific Ocular Imaging Society (APOIS) PCV Workgroup, and analyzed the accuracy of diagnosing PCV based on CFP or OCT features^12^. The authors noted that extensive subretinal hemorrhage and orange nodules, both CFP features graded by retinal specialists, had a sensitivity of 62–69% and AUC of 60–74%, less than the three major OCT criteria (sub-RPE ring like lesion, enface OCT complex RPE elevation, sharp-peak PED)^10^. Furthermore, another recent study by Xu et al. also used only their CFP-based model to differentiate PCV from AMD, with an accuracy of 75%^16^. Accordingly, our CFPs-based model alone achieved a valid performance comparable with that of earlier studies.

With regard to OCT, Gemmy Cheung et al. evaluated the presence of three major OCT criteria for diagnosing PCV, and reported that achieved an 82% accuracy for three retinal specialists and trainees^12^. Xu et al. assessed the OCT-based model alone, which reached the accuracy of 83.2% and proposed a bi-modal DL model directly combining CFPs and OCT images with the improvement in accuracy to 87.4%^16^, which was comparable to our DL model performance in combination of CFPs and OCT biomarkers.

In the present study, we selected six specific OCT biomarkers, which not only included the biomarkers mentioned in APOIS but also the other biomarkers that had been commonly discussed in other literature. We believe that these biomarkers still play some roles in differentiatiing PCV and nAMD, and each biomarker may be associated with each other in some way. Therefore, we used MCA, a statistical method under the concept of extracting features from each categorical variable and transforming them into new components to reassemble all the OCT biomarkers into new principal components for further analysis.

At present, MCA has been used in numerous fields ranging from population studies to social sciences, ecology, market research, health, and particularly medicine^19–21^. Mathematically, the MCA approach can aid in defining a set of dichotomous variables into a multi-dimensional space by Euclidean distance. Compared with binary or nominal variables, MCA provides dimension reduction for nominal data by considering the association between several biomarkers. It extracts useful features for prediction by calculating eigenvalues and eigenvectors. This method can aid in applying images for the DL algorithm as well as adding clinical categorical biomarkers, converting the six binary OCT biomarkers into six continuous multiple correspondence components, for further development of an applicable AI model. The continuous variables can provide more information to improve prediction performance than the binary variables, especially the continuous variables that have important features extracted through the MCA method. To our knowledge, this is the first study exploiting MCA method in the field of AI in ophthalmology.

In our literature review, we noted that few studies used imaging-based DL models for diagnosing PCV. Yang et al. delivered ICGA images to public-available AutoML (https://cloud.google.com/automl, Google Inc., CA, USA) for model training^16^. Xu et al. proposed a bi-modal DL model, directly combining CFPs and OCT images, to classify AMD and PCV but with limited cases^17^. From a DL perspective, combining a CFP-based model with OCT-based biomarkers possesses many desired qualities and advantages as an applicable modality for differentiating PCV from nAMD. First, we obtain superior near-microscopic structural detail of the retina in vivo provided by OCT. Second, these two exam approaches are more easily available and noninvasive than FA or ICGA. Third, with the progress in the ability of computer calculations and the multidisciplinary model of AI, combining information from different assessments became the art of science. Fourth, the specific biomarkers, but not entire OCT images, provide diagnostic criteria with high sensitivity and specificity for PCV^12^.

There are some limitations in our study. The primary constraint is the limited data, which was often encountered in a single-center study. Nonetheless, we enrolled nearly 700 cases, considered relatively enough for a training DL model. We also performed five-fold cross-validation to prevent overfitting of the model in the finite dataset, and the results achieved robust performance. As for classifying another type of AMD, it will be studied in future research. The secondary limitation is the B-scan spectral-domain OCT, which only demonstrated a central cross-sectional view of the macula, possibly missing the detection of the principal lesions of PCV. Hence, the actual OCT biomarkers in the PCV group must be more than that annotated. Thus, B-scan spectral0domain OCT displayed potentially favorable accuracy to classify PCV in the real-world setting. Third, we used MCA-transformed categorical OCT biomarkers, rather than direct OCT images, to combine with CFPs. Because of the complexity and difficulty of the DL design in direct combination with two different images, the present study provides a novel aspect of bi-modal DL training using an ensemble stacking technique in a combination of CFP-based model and MCA-transformed OCT biomarkers. MCA, which transformed any type of clinical categorical features into continuous components, provides another option for further DL training. This method helps us to fuse any type of categorical information into a DL algorithm for further ensemble stacking technique. It provides us more intuitive and explainable approach to develop a supervised learning system. Fourth, the labelling of OCT biomarkers would be influenced by an inspector’s individual perception. The experienced retinal specialist can decrease the variance as much as possible. In the future, auto-detection of OCT biomarkers by AI will be the next step for differentiation of nAMD and PCV.

In conclusion, this study proposed a new AI model using ensemble stacking technique, which combined a CFP-based DL model and OCT-based biomarkers, for differentiating between PCV and nAMD. This model achieved a robust performance and favorable accuracy by training nearly 700 active cases with suitable imaging quality and transfer learning architecture. Furthermore, we introduced MCA, a novel technique of transforming categorical data into principal components, to handle the dichotomous data for combining them with another image DL system. This work could offer an alternative method of developing a multi-modal DL model, improve its efficiency for distinguishing different diseases, and assist the broad application of medical engineering in a DL model design. This novel approach may have a transformative effect on distinguishing different diseases and potential importance as a routine diagnostic measure to promote wider application of medical engineering in a DL model design.

## Methods

### Data set

We retrospectively examined 1100 eyes in 1100 cases with active nAMD or PCV (800 eyes with nAMD and 300 eyes with PCV) from January 2012 to June 2017 at Taipei Veterans General Hospital (TVGH) using synchronous CFPs and OCT images in specific eyes. The selected images were captured with dilated pupil, and then assessed through serial image examinations. All the cases were diagnosed as per the standard using CFPs (CX-1, Canon Components Inc., Japan), OCT (RTvueXR, Optovue, Fremont, CA), FA, and ICGA (Heidelberg Engineering Inc., Heidelberg, Germany). Four retinal specialists (Y-B C, Y-M H, D-K H, and A-F L) confirmed the image finding and diagnosis. The PCV cases conformed to the EVEREST criteria^19^, whereas typical nAMD was considered as type 1, 2 or 3 choroidal neovascular (CNV) networks without aneurysmal or polypoidal alternations in FA or ICGA. The OCT images were further reviewed and labeled for the presence of six specific biomarkers by an experienced retinal specialist (Y-B C), including double-layer sign, triple-layer sign, thumb-like PED, notch PED, M-shape PED, and ring/bubble sign (including strings-of-pearl sign)^1^. Patients with poor image quality, extra-macular PCV, and concurrent retinal diseases such as diabetic retinopathy, vitreo-macular interface disease, retinal vein or artery occlusion, myopic CNV, or CNV secondary to infectious or inflammatory diseases were excluded from the study.

If the diagnosis or classification was uncertain or disagreed between different retinal specialists, the images would not be enrolled for further model training. After the classification of nAMD or PCV by using multi-image modality, including ICGA, and monitoring image quality, all the CFPs and OCT images of suitable quality were separated into these two groups for further model training. In the nAMD group, 419 cases (85%) were assigned to the training set and 72 cases (15%) to the testing set. In the PCV group, 182 cases were assigned to the training set, and the other 26 cases were assigned to the testing set.

This study was approved by the Institutional Review Board of Taipei Veterans General Hospital and conformed to the tenets of the Declaration of Helsinki (2017-10-006AC). Informed consent was waived by the ethics committee of Taipei Veterans General Hospital because data were de-identified and decoded.

### Model training

All the images were preprocessed and trained by Institute of Statistics, National Chiao Tung University, Taiwan. Figure 1 reveals the framework of model training by combining CFPs and OCT biomarkers.

The first step was to train EfficientNet^18^ by CFPs to obtain the prediction value. Google had published EfficientNet in 2019. It used relatively fewer parameters to achieve better performance than previous models in ImageNet Large-Scale Visual Recognition Challenge (ILSVRC). Its validity verification also provides further support and credibility of the model in many datasets. EfficientNet includes multiple versions (B0- B7), and different versions demands different resolution of the images.

The second step was to transform the binary OCT biomarkers (including double-layer sign, triple-layer sign, notch PED, M-shape PED, and ring/bubble sign) into principal components using MCA. The last step is to use the ensemble stacking technique for the combination of the results from the two steps. This model is the first DL system designed for distinguishing PCV and nAMD by combining a CFP-based modality and OCT-based transformed features, rather than typical regression or pure image-based AI model. The description is illustrated step by step subsequently.

#### Preprocessing

##### Color fundus photographs (CFPs)

All the images were downloaded in a standard JPEG format according to the manufacturer’s setting. Any identifiable or diagnostic information was removed to avoid interference. The first step was to resize the CFPs resolution. We selected the EfficientNet B3 version as pre-trained model based on the computing resources with a 300 × 300 input shape. The next step was to standardize the CFPs input to z-score to confirm that all the images have constant intensity and contrast. The model structure of EfficientNet-B3 was maintained except for replacing the fully connected layers with a binary output layer, the Softmax function. Besides, the weight of EfficientNet B3 was discarded and retrained by the training images from the TVGH dataset. The detail and value of the hyperparameters are listed in Supplementary Table S1.

##### Optical coherence tomography (OCT) biomarkers

After the OCT biomarkers were labeled by a retinal specialist and recorded in categorical format, which was 0 (negative) and 1 (positive), we transformed the data using MCA, a statistical method of converting categorical data into continuous values in a matrix format. After the transformation by MCA, the more essential components will be selected as features for improving the predicted performance.

#### Imbalanced data

Imbalanced data can cause severe problems in intelligent medical diagnosis, especially increasing the bias in model training. Two methods were used in this study to resolve this problem, weighted binary cross entropy (WBCE) and synthetic minority oversampling technique (SMOTE).

##### Weighted binary cross entropy (WBCE)

Cost-sensitive learning methods can improve the problem of imbalanced data. This approach set different weights for each classification on the basis of the ratio between the majority and minority group.

Songqing et al. attempted to use the method with weighted softmax on malware image classification. They aimed to minimize the loss value with WBCE and performed calculations using a specific formula^20^. It changed the weight of the minority (PCV group in this study) in binary classification.

##### Synthetic minority oversampling technique (SMOTE)

During the process of model training, several hyperparameters must be tuned. The imbalanced data ratio will lead to model bias in the training process and increase the difficulty of hyperparameter selection. SMOTE is an oversampling approach that synthesize new images by linear interpolation to reduce the problem caused by imbalanced data^22,23^.

#### Five-fold cross-validation

Cross-validation is a robust method to reduce the sampling bias. In this study, the training dataset was split into five subsets as the validation sets by turns. One of them was selected as the “validation set” and the remaining four were “training sets”. The average of the accuracy in the five validation sets was used as the standard criterion for the selection of hyperparameters; those variables then determined the structure of CNN. Figure 5 demonstrates how the training dataset split into five subsets.

#### Multiple correspondence analysis (MCA)

MCA is based on the concept of dual scaling, which captures either linear or nonlinear relationships equally well, and works well with categorical variables, especially when each variable possesses some degree of connection with each other. It is a multivariate method that distributes values of relative frequency in specific dimensional space and then applies the distance between variables to establish the degree of similarity of variables in a matrix format. In this study, we used MCA to distribute the categorical variables of six OCT biomarkers and transform them into continuous principal components in a matrix format for further feature engineering and model training. The number of principal components was a hyperparameter selected by the best performance of the validation set.

#### Feature engineering

**Figure 6:**
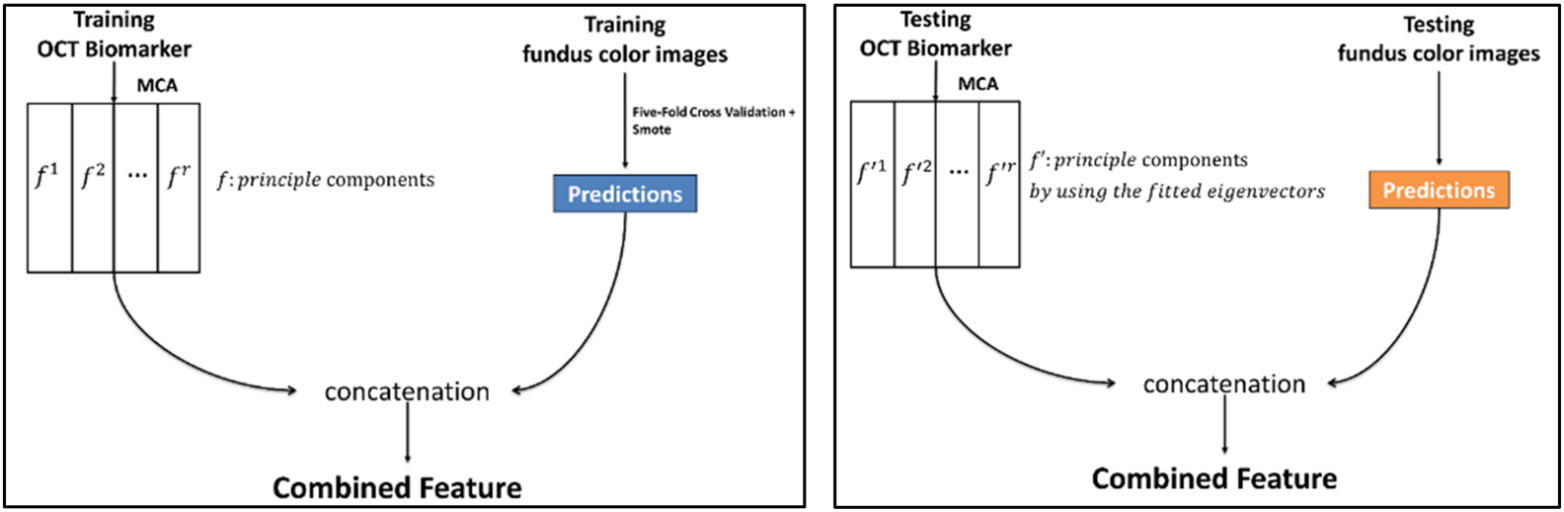
Feature engineering. This figure revealed the process of feature engineering in OCT biomarkers and CFPs with training (left) and testing (right) datasets.

In this step, feature engineering included CFPs, OCT biomarkers, and their combination. First, the data were split into training and testing sets. The prediction value represented the possibility of PCV based on CFPs by EfficientNet-B3. The OCT biomarkers were transformed into principal components in a matrix format by MCA. The concatenation of feature extractions from CFPs and OCT biomarkers provided the combined information. In the testing dataset, when transforming the OCT biomarkers into principal components, we used eigenvectors that were also fit from the training dataset. Figure 6 demonstrates the feature engineering for the combination of CFPs and OCT biomarkers.

#### Ensemble stacking technique

**Figure 7:**
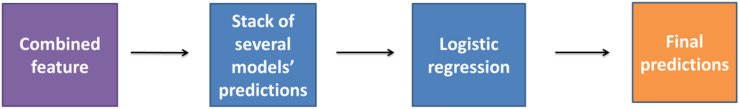
Ensemble stacking technique. It was used to combine two different information from separate feature engineering, CFPs and OCT biomarkers, and then to choose differernt learning models (including XGBoost, LightGBM, and CatBoost) to create new features by stacking technique for final prediction.

Stacking is a machine learning technique of using different models to obtain the prediction as a new feature. It is such an ensemble learning model to provide the best combination from multiple models. Figure 7 demonstrates how the stacking technique works in this study. First, we used the combined features from CFPs and OCT biomarkers, and chose several machine learning models (including XGBoost, LightGBM, and CatBoost) to identify the best validation for stacking their predictions in each of them. Next, forecasts from each model were considered new features, which then underwent logistic regression for final prediction.

## Supplementary Information

**Supplementary Tables.**
