# Supplementary material for: Deep learning and ensemble stacking technique for differentiating polypoidal choroidal vasculopathy from neovascular age-related macular degeneration
Source: Sci Rep. 2021 Mar 30;11:7130. doi: 10.1038/s41598-021-86526-2 (PMC8010118; doi:10.1038/s41598-021-86526-2)
Supplement: Supplementary file 1 — Supplementary Tables. [file 41598_2021_86526_MOESM1_ESM.docx]

| **Model** | **EfficientNet-B3** |
| --- | --- |
| **Hyperparameters** | |
| Optimizer | Adam |
| Epoch | 15 |
| Batch | 10 |
| The Beta in Weighted Binary Cross Entropy | 10 |
| **The Performance of Five-Fold Cross Validation** | |
| Average accuracy | 0.8070 |
| Standard deviation of accuracy | 0.0076 |
| Average AUC | 0.8355 |
| **Supplementary Table S1. The Detail of Hyperparameters in the EfficientNet-B3 pre-trained Model and the Performance of Five-fold Cross Validation.** | |

| The AUC of different hyperparameters R on the validation set of MCA components | |
| --- | --- |
| R=1 | 0.6980 |
| R=2 | 0.6954 |
| **R=3** | **0.7034** |
| R=4 | 0.7002 |
| R=5 | 0.7021 |
| The AUC of different hyperparameters R combined with CFPs in test set | |
| R=1 | 0.8825 |
| R=2 | 0.8916 |
| **R=3** | **0.8857** |
| R=4 | 0.8841 |
| R=5 | 0.8649 |
| **Supplementary Table S2. The AUC of different hyperparameters on MCA components and its combination with CFPs in test set.** | |
